# Legitimacy of work tasks, psychosocial work environment, and time utilization among primary care staff in Sweden

**DOI:** 10.1080/02813432.2019.1684014

**Published:** 2019-11-04

**Authors:** Eva Anskär, Malou Lindberg, Magnus Falk, Agneta Andersson

**Affiliations:** a Department of Medical and Health Sciences, Division of Community Medicine, Linköping University, Linköping, Sweden;; b Primary Health Care Center in Mantorp, and Department of Medical and Health Sciences, Linköping University, Mantorp, Sweden;; c 1177 Medical Advisory Service, Linköping, Sweden;; d Research and Development Unit in Region Östergötland and Department of Medical and Health Sciences, Linköping University, Linköping, Sweden

**Keywords:** Primary care, occupational health, organization and administration, professional roles

## Abstract

**Objective:** Primary care staff faces a complex work environment including a heavy administrative work load and perceive some work tasks as illegitimate. This study aimed to elucidate associations between the perceived legitimacy of work tasks, the psychosocial work environment, and the utilization of work time among Swedish primary care staff.

**Design and setting:** The study was designed as a multicenter study involving all staff categories, i.e. registered nurses, primary care physicians, care administrators, nurse assistants and allied professionals, at eleven primary care centers in Sweden.

**Subjects:** Participants completed the Bern Illegitimate Tasks Scale and the Copenhagen Psychosocial Questionnaire. They also recorded time spent on all work tasks, day by day during two separate weeks.

**Main outcome measures and results:** More than a quarter (27%) of primary care physicians perceived a high proportion of unnecessary work tasks. After adjusting for profession, age and gender, the perception of having to perform unreasonable work tasks was positively associated with experiencing role conflicts and with the proportion of organization-related administration and service work tasks.

**Conclusion:** Across all staff groups, the perception of unreasonable work tasks was more pronounced among staff with a high proportion of non-patient related administration. Also, the perception of having to perform a large amount of illegitimate work tasks affected the psychosocial work environment negatively, which might influence staffs perception of their professional roles. These results illuminate the importance of decision makers to thoroughly consider the distribution and allocation of non-patient related work tasks among staff in primary care.

**Key points**
We observed an interaction between perception of having a large proportion of illegitimate work tasks and impaired psychosocial work environment.
• More than a quarter of the primary care physicians perceived a high proportion of unnecessary work tasks.
• Across all staff groups, performing unreasonable work tasks was associated with an experience of having role conflicts.
• Across all staff groups, a perception of performing unreasonable work tasks was associated with the proportion of non-patient related administrative work tasks.

## Introduction

Efficient use of health care resources is as crucial as it is fundamental. In general, primary health care tend to be a demanding, multifaceted work place. The staff faces a complex work environment [1,2], often with a heavy administrative work load [3,4], not least in Sweden. Osborn et al. reported that, after the US, which is the only one of the ten countries studied without a national health system, Swedish primary care physicians (PCPs) were the most dissatisfied with the health care system. In Sweden, only 19% of PCPs stated that the system worked well; whereas 27% of PCPs in Germany and 67% in Norway stated that their systems worked well [4]. Swedish PCPs have also reported to experience negative psychosocial work conditions more frequently than other professions in primary care [5] and Kuusio et al. concluded that reducing psychosocial demands may contribute to better organizational commitment among PCPs [6]. Teles at al. noted that adverse psychosocial working conditions were associated with a poor quality of life among primary care staff [7].

In Sweden, there is an ongoing debate concerning the efficient use of health care resources in meeting population demands and in delivering health care services. The number of staff in the Swedish health care sector has increased continuously and is to date proportionally larger per capita compared to most other European countries [8]. In spite of this, the Swedish health care sector struggles with issues of poor accessibility and long waiting times for diagnosis and treatment, both at hospitals and in primary care [8,9]. During recent decades, administrative work tasks have been gradually redistributed from administrators to medical staff, possibly indicating that available medical resources are not being used efficiently [10].

A recent investigation performed by the Swedish government concluded that the amount of administrative work tasks has increased in the health care sector. Work tasks that are administrative in character include, for example, documentation and reporting information between different professionals and groups or organizing and coordinating activities [10]. The trend of reducing the numbers of care administrators and nurse assistants (NAs) has contributed to the current situation, where PCPs and RNs perform proportionally more administrative work tasks than before [10]. This, in turn, has reduced the amount of time they have for direct patient work, which is in general perceived as more meaningful, according to Bringsén et al. [11]. Accordingly, administrative work tasks that do not require medical education or skills may be perceived as illegitimate by medical professionals. The concept of illegitimate work tasks has been described in several previous studies [12–14]. These tasks are generally divided into two facets; *unnecessary work tasks*, defined as work tasks that should not be necessary at all, provided that things were better organized; and *unreasonable work tasks*, defined as work tasks beyond the specific job description of a given person. A survey in Sweden showed that among primary care physicians, work stress was associated with a perception of having to perform illegitimate work tasks [15]. To our knowledge, no previous study has investigated the perceived legitimacy of work tasks and its association with the psychosocial work environment also for other professions in primary care in Sweden.

## Aim

The present study aimed to elucidate associations between the perceived legitimacy of work tasks, the psychosocial work environment, and the utilization of work time among Swedish primary care staff.

## Methods

### Setting and participants

Eighty percent of Swedish health care is publicly funded and delivered by the county councils [8]. The health care organization in Sweden consists of 21 county councils, and all provide both hospital care and primary care. Thirteen of the county councils have an extended responsibility for regional development, and therefore, they are classified as Regions. The total number of primary care centers in Sweden is about 1200 [16]. The present multicenter study was performed in southeast Sweden and included primary care centers from four county councils (Östergötland, Jönköping, Kalmar, and Södermanland). Based on purposive sampling, we contacted the managers of 23 primary care centers. The goal was to capture a wide range of perspectives, including different center sizes, geographical locations, and urban and rural settings. The study was approved by the managers of 11 primary care centers, five rural and six urban. The largest had 81 employees and the smallest had 20.

All professionals at the primary care centers (n = 441) were invited to participate, including RNs (38%), PCPs (25%), care administrators (17%), NAs (10%), and allied professionals (10%) (physiotherapists, occupational therapists, psychologists, counselors, dieticians, and chiropodists). Data collection was carried out from March 2014 to February 2015. The employees received oral and written information about the study.

### Data collection

A questionnaire was distributed by e-mail to all staff members at each primary care center, with Publech^®^ Survey 5.7 software. One reminder was sent after two weeks. The first section of the questionnaire consisted of introductory questions regarding gender, profession, and the number of years in the profession. The participants were also asked to estimate the proportion of time spent on work tasks involving patients and the proportion spent on other work. The results of those estimations were reported in an earlier publication by Anskär et al. [5]. The second section of the questionnaire consisted of the Bern Illegitimate Tasks Scale (BITS) [12–14] and the Copenhagen Psychosocial Questionnaire (COPSOQ) [17–21]. The staff was also invited to participate in a time study, described below.

### Illegitimacy of work tasks

The BITS questionnaire for assessing illegitimate work tasks was previously tested for reliability [13,14] and validated, with satisfactory results [22]. It was also previously used in a Swedish health care setting [15]. Some items assessed unnecessary work tasks (items 1-4); for example: ‘Do you have work tasks to take care of, which keep you wondering if they have to be done at all?’ Other items assessed unreasonable work tasks (items 5-8), for example: ‘Do you have work tasks to take care of, which you believe should be done by someone else?’ All items had five response options, ordered on a Likert-type scale, ranging from 1 (never), to 5 (frequently) [14,15,22]. Higher scores corresponded to a higher degree of illegitimacy perceived for work tasks.

### Psychosocial work environment (PWE)

PWE was measured with six scales selected from the COPSOQ instrument, which had been validated and reliability tested [21]. The six scales included quantitative demands (4 items), stress (4 items), role conflicts (4 items), quality in work (3 items), conflicts between work and personal life (4 items), and a positive impact of work on personal life (2 items). The two scales ‘quality in work’ and ‘positive impact of work on personal life’ were not part of the original COPSOQ, but they were added by the creators of the COPSOQ for inclusion in studies conducted in the health care sector. The total score on each COPSOQ scale was calculated as the mean of the scores for the individual items in that scale. High scores on the scales ‘quantitative demands’, ‘stress’, ‘role conflicts’, and ‘conflicts between work and personal life’ indicated a negative psychosocial work environment. High scores on the scales ‘quality in work’ and ‘positive impact of work on personal life’ indicated a positive psychosocial work environment.

### Time study

A time study was conducted with a self-reporting form that was developed specifically for this study. Participants used the form to record the time they spent on work tasks day by day over two separate weeks. The form contained three main categories and a number of subcategories for each main category. The first main category was *direct patient-related work tasks*; the second main category was *indirect patient-related work tasks*; and the third main category was *other work tasks* [5].

Administrative work tasks can be defined as reporting information between different principals and managers, coordinating activities, and organizing systems. A number of work tasks that were administrative and/or service-oriented were selected from the two main categories: indirect patient-related work tasks and other work tasks. The selected work tasks were organized into two new categories: *Patient-related administrative work tasks* and *Organization-related administrative and service work tasks* (Box 1).

**Box 1.** Administrative work tasks.

**Patient-related administrative work tasks**	**Organization-related administration and service work tasks**
Documentation	Meetings at the work place
Dictation	Other writing tasks/administration
Administering appointments	Managing equipment and facilities
Signing journal entries	E-mail management
Referral management	Meetings outside the work place
Managing mail	Scheduling
Prescribing medical drugs	Managing computer problems
Entering data into health care records and quality registries	Ordering medical supplies, including laundry
Prescribing medical aids	Non-patient-related telephone communications

### Statistical analyses

The two facets of the BITS (unnecessary work tasks and unreasonable work tasks) were dichotomized into scores above or below a cut-off value that distinguished between perceived legitimate (below the cut-off) and illegitimate (above the cut-off) work tasks, based on Aronsson et al. (cut-off value = 3.5 for both facets) [15]. Descriptive statistics were used to calculate the distributions of staff members with scores above or below the cut-off values for illegitimate work tasks in both facets of the BITS. Chi-square tests were used to analyze the significance of differences between professions in their responses to items in both facets. Descriptive statistics were used to calculate the proportions of different work tasks.

Logistic regression was used to evaluate the association between perceived unnecessary work tasks and the COPSOQ scales; the association between perceived unreasonable work tasks and COPSOQ scales; the association between perceived unnecessary work tasks and the proportions of different work tasks; and the association between perceived unreasonable work tasks and the proportions of different work tasks. Analyses were adjusted for profession, age, and gender. The results from the logistic regressions were expressed as odds ratios (OR) with 95% confidence intervals (CI).

## Results

Of the 441 individuals invited to participate in the study, 391 agreed to participate in some or all parts of the study (Table 1). In all staff categories, 88 to 100% of participants were women, except PCPs, where 55% were women and 45% were men.

**Table 1.** Study sample, response rates, categorized by profession, age, gender and study section.

	Study sample				Respondents					Questionnaire BITS^a^		Questionnaire PWE^b^		Time study	
Professions	*N*	(%)	*n*	(%)	Mean age^c^	(min–max)	(SD)	♀*n*	(%)	*n*	(%)	*n*	(%)	*n*	(%)
Registered nurse	166	(38)	148	(38)	52	(22–67)	(9.6)	142	(96)	127	(86)	127	(86)	139	(94)
Physician	109	(25)	86	(22)	46	(28–70)	(11.7)	47	(55)	63	(73)	63	(73)	75	(87)
Care administrator	75	(17)	70	(18)	49	(26–66)	(11.2)	70	(100)	65	(93)	65	(93)	61	(87)
Nurse assistant	46	(10)	44	(11)	54	(33–67)	(8.7)	44	(100)	35	(80)	35	(80)	42	(96)
Allied professionals	45	(10)	43	(11)	47	(24–65)	(12.4)	38	(88)	40	(93)	39	(91)	33	(77)
Total sample (All professions)	441	(100)	391	(100)	50	(22–70)	(10.9)	341	(87)	330	(84)	329	(84)	350	(90)

a Bern Illegitimate Tasks Scale, for measuring perceived illegitimate work tasks.

b Copenhagen Psychosocial Questionnaire, for measuring psychosocial work environment.

c Did not add up to total sample, due to internal drop out, *n* = 337.

More than a quarter (27%) of PCPs scored above the cut-off value for BITS regarding unnecessary work tasks, which was significantly more (*p* < .001) than the proportion observed in all other professions in the survey. For both PCPs and RNs, 8% scored above the cut-off value regarding unreasonable work tasks. None of the NAs scored above the cut-off value for unreasonable work tasks. Ten participants scored above the cut-off values for both unnecessary and unreasonable work tasks. These participants included five physicians, four RNs, and one allied professional (Table 2).

**Table 2.** Number of staff members that perceived illegitimate work tasks above the cut-off values for unnecessary and unreasonable work tasks.

	Illegitimate work tasks (BITS)			
		Unnecessary work tasks above the cut-off value^a^	Unreasonable work tasks above the cut-off value^b^	Unnecessary and unreasonable work tasks above the cut-off values^a^^,b^
Professions	*n*	*n* (%)	*n* (%)	*n* (%)
Registered nurse	127	12 (9)	10 (8)	4 (3)
Primary care physician	63	17 (27)	5 (8)	5 (8)
Care administrator	65	3 (5)	1 (1.5)	0 (0)
Nurse assistant	35	2 (6)	0 (0)	0 (0)
Allied professionals	40	2 (5)	1 (3)^c^	1 (2.5)
Overall	330	36 (11)	17 (5)	10 (3)

a A large proportion of unnecessary work tasks to a high degree.

b A large proportion of unreasonable work tasks to a high degree.

c Calculated with 39 participants, due to participant drop out from this professional category.

Across all staff groups, a perception of having illegitimate work tasks was significantly associated with low self-reported PWE scores. We also found a positive association between perceived role conflicts and scores above the cut-off for unreasonable work tasks (OR 1.11, odds increased with 11% for every score point higher on the role conflict scale); i.e. higher frequencies of perceived unreasonable work tasks were associated with higher frequencies of role conflicts, stress was also significantly positively associated with the perception of unreasonable work tasks (OR 1.06, odds increased with 6% for every score point higher on the stress scale). Moreover, the perception of having unnecessary work tasks was significantly positively associated with role conflicts (OR 1.07). In contrast, quality in work was significantly negatively associated with unnecessary work tasks (OR 0.94); i.e. higher work quality corresponded to a lower frequency of perceived unnecessary work tasks. Similarly, quality in work was significantly negatively associated with unreasonable work tasks (OR 0.95); i.e. higher work quality corresponded to lower frequencies of unreasonable work tasks (Table 3).

**Table 3.** Associations between illegitimate work tasks (scores above the cut-off value) and the psychosocial work environment (*N* = 329).

	Illegitimate work tasks (BITS)							
	Unnecessary work tasks (above cut-off value)				Unreasonable work tasks (above cut-off value)			
Psychosocial work environment (COPSOQ)	*n*^a^	OR	(95 % CI)	*p*-value	*n*^a^	OR	(95 % CI)	*p*-value
Role conflicts^b^	325	1.07	(1.05–1.098)	<.001	324	1.11	(1.06–1.16)	<.001
Quantitative demands^b^	328	1.03	(1.01–1.05)	.002	327	1.03	(1.01–1.06)	.017
Stress^b^	325	1.04	(1.02–1.06)	<.001	324	1.06	(1.03–1.09)	<.001
Quality in work^c^	326	0.94	(0.91–0.97)	<.001	325	0.95	(0.91–0.99)	.019
Conflict between work and personal life^b^	325	1.02	(1.01–1.04)	<.001	324	1.04	(1.02–1.06)	<.001
Positive impact of work on personal life^c^	325	0.99	(0.971–0.999)	.035	324	1.00	(0.98–1.02)	.93

OR: odds ratio; CI: confidence interval; COPSOQ: Copenhagen Psychosocial Questionnaire; Regression analyses were adjusted for profession, age, and gender.

a Numbers may not reflect the total, due to participants dropping out.

b A low value is a positive rating.

c A high value is a positive rating.

We found that a high BITS score, across all staff groups, for perceived unreasonable work tasks was significantly negatively associated with the proportion of self-reported direct patient-related work tasks (OR 0.93); i.e. a higher frequency of direct patient-related work tasks corresponded to a lower frequency of unreasonable work tasks (Table 4).

**Table 4.** Associations between illegitimate work tasks above the cut-off value and different work tasks (*N* = 290).

	Illegitimate work tasks (BITS)							
	Unnecessary work tasks (above cut-off value)				Unreasonable work tasks (above cut-off value)			
Self-reported work tasks	*n*^a^	OR	(95 % CI)	*p*-value	*n*^a^	OR	(95 % CI)	*p*-value
Direct patient-related work tasks	280	0.98	(0.95–1.01)	.216	279	0.93	(0.89–0.98)	.003
Indirect patient-related work tasks	286	1.01	(0.98–1.05)	.463	285	1.02	(0.95–1.08)	.659
Other work tasks	288	1.00	(0.98–1.03)	.805	287	1.04	(1.01–1.07)	.012

OR: odds ratio; CI: confidence interval; Regression analyses were adjusted for profession, age, and gender.

a Numbers may not reflect the total, due to participants dropping out.

We also found that high BITS scores, across all staff groups, for perceived unreasonable work tasks were significantly positively associated with a self-reported high proportion of organization-related administration and service work tasks (OR 1.05); i.e. higher frequencies of self-reported organization-related administrative and service work tasks corresponded to higher frequencies of unreasonable work tasks (Table 5).

**Table 5.** Associations between illegitimate work tasks above the cut-off value and administrative and service work tasks (*N* = 290).

	Illegitimate work tasks (BITS)							
	Unnecessary work tasks (above cut-off value)				Unreasonable work tasks (above cut-off value)			
Self-reported work tasks	*n*^a^	OR	(95 % CI)	*p*-value	*n*^a^	OR	(95 % CI)	*p*-value
Patient-related administrative work tasks	283	1.02	(0.98–1.06)	.335	282	0.98	(0.90–1.07)	.695
Organization-related administration and service work tasks	287	1.01	(0.98–1.04)	.395	286	1.05	(1.01–1.08)	.007
Total administration and service work tasks	282	1.04	(1.001–1.07)	.046	281	1.04	(1.00–1.09)	.082

OR: odds ratio; CI: confidence interval; Regression analyses were adjusted for profession, age, and gender.

a Numbers may not reflect the total, due to participants dropping out.

## Discussion

This study aimed to investigate associations between the perceived legitimacy of work tasks and psychosocial environment among primary care staff in Sweden. We also explored potential associations between the perceived legitimacy of work tasks and work time utilization. More than a quarter (27%) of PCPs scored above the cut-off for perceived unnecessary work tasks, which was a significantly higher proportion than that observed for all other professions. This result was consistent with the finding from our previous study that PCPs perceived more negative PWE than the other staff members [5]. Also, Osborn et al. found that Swedish PCPs were dissatisfied with the time available for each patient and with the health care system [4]. Semmer et al. stated that, the perception of a work task as a waste of time or as something that should be performed by someone else could be considered a sign of low respect for the person that is expected to perform the task. They also stated that illegitimate work tasks can negatively affect a staff’s professional identity [14]. Moreover, illegitimate work tasks were shown to be associated with experiencing role conflicts and could potentially violate established norms for the types of work tasks that can be expected from an employee [14]. These concepts correspond to the results presented in this study; i.e. perceptions of illegitimate work tasks were associated with an unfavorable PWE, indicating that staff member competence was not being used effectively. Semmer et al. also points out that illegitimate work tasks might cause counterproductive work behavior. They pointed out the importance of assigning appropriate work tasks to different coworkers to reduce the risk of inciting resentment and role conflicts [14]. An example of a role conflict, described by Spehar et al., can be between the clinical and leadership roles among general practitioners [23]. Perceiving this kind of conflict, due to the leadership role, might partly explain the negative PWE among PCPs [5].

The governmental investigation regarding Swedish primary care performed in 2016 emphasized the fact that the administrative burden in Swedish primary care has increased [10]. Staff members, i.e. all medical staff, in primary care reported that a great proportion of work time did not involve directly working with patients [5]. The results of the present study suggest that, for all staff groups, a heavy administrative work load may contribute to a perception of having to perform unnecessary work tasks. These results were consistent with earlier findings, which showed that PCPs in Sweden spent proportionally more of their work time on administration compared to their colleagues in other countries; for example, in Germany, the Netherlands, Norway, and Switzerland [24]. In our survey, when staff members had self-reported a large proportion of other work tasks (e.g. meetings, e-mail management, scheduling, and writing tasks/administration), it was associated with a high score for unreasonable work tasks. Conversely, when staff members reported a greater proportion of direct patient-related work tasks, it was associated with a lower score for unreasonable work tasks. This result was consistent with those of Bringsén et al., who noted that the closer a work task was to working directly with patients, the more meaningful it was perceived by health care staff [11], as well as with those of Halvorsen et al., who showed that dealing with common symptoms, chronic somatic diseases, and psychiatric diseases were examples of work tasks perceived as meaningful among primary care staff [25]. Our results are also is in line with Areskoug Josefsson et al., who found that working with patients contributes to joy at work [26].

Our results elucidate the importance of ensuring that all medical staff has a balance between direct patient-related work tasks and non-patient and service-related administration work tasks. However, there are many different types of administrative work tasks. Some administrative work tasks, especially patient-related administrative tasks, by necessity, must be performed by medical professionals, and in our study these work tasks did not affect the perception of illegitimate work tasks. This finding corresponds to another result in our study, which showed that indirect patient-related work tasks, for example documentation in health care records, did not increase the perception of illegitimate work tasks. Documentation is a valuable work tool and very important in securing patient safety. Another example of an indirect patient-related work task is to write out sick leave certifications. This task requires a large proportion of work time among PCPs in primary care. Ljungquist et al. stated that PCPs perceived this work task as more problematic than physicians in other specialties [27]. However, despite this perception, we found no significant association between illegitimate work tasks and indirect patient-related work tasks.

The work relationships among staff members are essential, both for the PWE and for effective work production. Work tasks must be assigned to staff with the required competencies and skills [28–30]. As a strategy for promoting well-being among general practitioners, Hall et al. asserted that increasing resources with more administrative staff, may benefit the well-being of staff members [31]. Taken together, these findings indicated that non-patient administrative and service-related work tasks would best be assigned to care administrators or service personnel.

## Strengths and limitations

The primary strengths of this study were the overall high response rate and the wide variety of primary care center sizes and geographical locations. The validated instruments, BITS and COPSOQ, also strengthened the credibility of the study results. The cut-off values for the BITS facets have been used in Swedish settings previously.

The design of the time study, which required recording data every hour, minimized the risk of recall bias. The self-reporting method might have carried some methodological challenges; e.g. the interpretation of work task definitions may have varied among participants. To avoid this problem, participants were informed which tasks were designated direct patient-related, indirect patient-related, and other work. In addition, each participant received a pamphlet with instructions on how to complete the time study form.

One limitation was that, out of the 23 managers that were contacted, only 11 of them approved participation in the study; this low rate may have affected the results. Another study limitation was that the form used in the time study was constructed by the authors specifically for this study, and it was not tested previously. However, it was validated by two experts, both with long experience in primary care.

## Conclusions

The perception of unreasonable work tasks was more pronounced among staff with a high proportion of non-patient related administration. Also, the perception of having to perform a large amount of illegitimate work tasks affected the psychosocial work environment negatively, which might influence staffs perception of their professional roles. These results illuminate the importance of decision makers to thoroughly consider the distribution and allocation of non-patient related work tasks among staff in primary care.

## Data Availability

The datasets used and analyzed during the current study are available from the corresponding author on reasonable request.
